# Post-traumatic fulminant paradoxical fat embolism syndrome in conjunction with asymptomatic atrial septal defect: a case report and review of the literature

**DOI:** 10.1186/1752-1947-5-142

**Published:** 2011-04-10

**Authors:** Franz Mueller, Christian Pfeifer, Bernd Kinner, Carsten Englert, Michael Nerlich, Carsten Neumann

**Affiliations:** 1 Regensburg University Medical Center, Department of Trauma and Orthopedic Surgery, 93042 Regensburg, Germany

## Abstract

**Introduction:**

Fat embolism syndrome with respiratory failure after intramedullary nailing of a femur fracture is a rare but serious complication in trauma patients.

**Case presentation:**

We present the case of a 20-year-old Caucasian man who experienced paradoxical cerebral fat embolism syndrome with fulminant progression after intramedullary nailing of a femur fracture, in conjunction with a clinically asymptomatic atrial septal defect in a high position resulting in a right-to-left shunt.

**Conclusion:**

Fat embolism syndrome may occur as a fulminant complication following femoral fracture repair in the presence of a concomitant atrial septal defect with right-to-left shunt. Thus, in patients with cardiac right-to-left shunts, femurs should not be nailed intramedullary, not even in cases of isolated injuries.

## Introduction

Fat embolism is caused by bone marrow components, in the form of cell debris and yellow bone marrow, entering into the systemic circulation and into the parenchyma of the lungs via the venous sinus [1]. Fat embolism syndrome (FES), however, is the symptomatic manifestation of fat embolism with symptoms such as respiratory failure, thrombocytopenia or cerebral confusion [2], which occur within 48 hours after trauma in most patients [2,3]. The occurrence of FES after intramedullary nailing of femur fractures is a rare but dreaded complication. Therefore, the application of an external fixation as an initial treatment is particularly recommended for multiple-trauma patients. However, scientific evidence from prospective multi-center studies is still required in order to validate this treatment in comparison with direct intramedullary nailing. Moreover, it also is unclear whether intramedullary nailing should be performed by reaming the medullary cavity. Many cases of fat embolism are known to proceed in a mild form showing few clinical symptoms. However, if cardio-respiratory volume is restricted or additional disorders or injuries are present, fulminant progression of FES may occur.

## Case presentation

**Figure 1:**
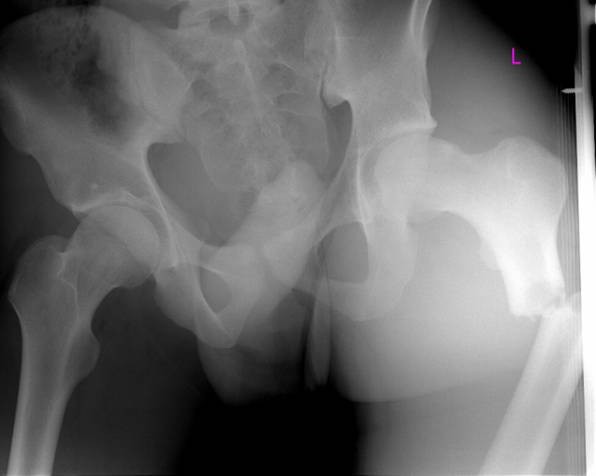
**Pre-operative radiograph of the pelvis showing proximal fracture of the left femoral shaft**.

**Figure 2:**
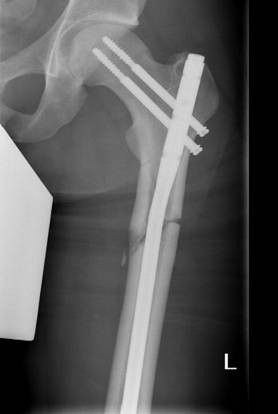
**Post-operative radiograph showing antegrade intramedullary nailing of the left femoral shaft**.

**Figure 3:**
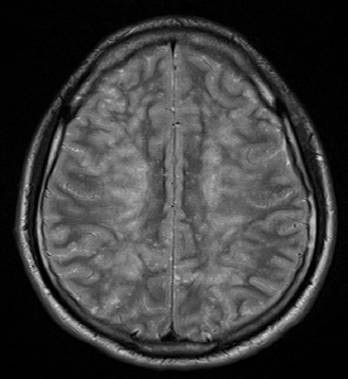
**MRI of the brain showing multiple lesions consistent with fat embolism**.

We present the case of a Caucasian man who experienced paradoxical cerebral FES with fulminant progression after intramedullary nailing of a femur fracture, in conjunction with a clinically asymptomatic atrial septal defect in a high position resulting in a right-to-left shunt, which is still present today. In spring 2008 our 20-year-old patient was driving a car, whilst wearing a seat belt, and collided head-on with a bus, and experienced trapping of his left leg. A Glasgow Coma Scale of 15 points and questionable initial unconsciousness were documented by the emergency medical services. After technical rescue operations our patient was hospitalized via air-bound transportation under analgo-sedation. Upon arrival in our emergency trauma room our patient was breathing spontaneously; he was awake and responsive and suffered from severe pain in the area of his left femur, which showed malpositioning. Due to the pain symptoms, our patient was initially intubated and mechanically ventilated. After that, the femur fracture was temporarily repositioned and fixed with a plaster. Diagnostic procedures were then performed, such as a whole body computed tomography (CT), showing a closed proximal fracture of his left femoral shaft (Figure 1), an ipsilateral type 2 open olecranon fracture and, as a secondary finding, a unilateral lung contusion. No other injuries could be detected; in particular the cerebral and abdominal CT scans were inconspicuous. Thus, the therapeutic indication for the definite treatment of these two injuries was established. After the diagnostic examination, our patient was transferred to the intensive care unit and, six hours after the trauma, was relocated to the operating theatre. At first, closed repositioning and antegrade intramedullary nailing of the left femur (10 mm thick) was conducted in supine and extended position without reaming the medullary cavity, followed by open repositioning and tension-band osteosynthesis of the olecranon. The intramedullary pin was proximally fixed with two hip screws, and distally by means of two bolts (Figure 2). There were no abnormal intra-operative findings, particularly no circulatory instability, no decrease of oxygen saturation, and no temporary drop in arterial blood pressure. Since post-operative vigilance did not improve, a cerebral CT scan was conducted on the third post-operative day, followed by a magnetic resonance imaging (MRI) of the skull on the sixth postoperative day. These scans showed multiple lesions in the brain stem, in the cerebellum, and in the cerebral hemispheres, which were consistent with fat embolism (Figure 3). Electroencephalography findings showed a serious diffuse brain malfunction. Moreover, significantly impaired perfusion was detected without any indication for a diffuse axial trauma. Trans-esophageal echocardiography showed an atrial septal defect in a high position resulting in a right-to-left shunt, which had not been diagnosed before, as well as several perforations in the area of the inter-atrial septum. There was no evidence of thrombosis, and all valves were soft and competent. Deep vein thrombosis of the leg and any clotting in the vena cava or in the pelvic veins as possible causes were excluded by means of duplex ultrasonography. Due to increasing vigilance, accompanied by a merely spontaneous opening of the eyes and some movements of the extremities, a tracheotomy was conducted. On the eleventh post-operative day our patient, breathing spontaneously, was transferred to the neurological rehabilitation unit. Radiological examination showed good results with regard to both the surgically treated extremities and primary wound healing. After one post-operative year, our patient was discharged from hospital, and neurological rehabilitation was continued on an out-patient basis. At that time our patient was breathing spontaneously, and the tracheostoma had healed; he was awake and responsive but showed distinctive cognitive deficits, particularly with regard to speech. At almost two years post-operative, our patient still requires care because of tetraparesis; independent mobilization is not yet possible.

## Discussion

Fat embolism occurs frequently and can be detected by means of trans-esophageal echocardiography in more than 90% of patients suffering from fractures of the long bones [1]. On the other hand, the incidence of FES is considerably lower: in a study of 274 consecutive patients with isolated femoral shaft fractures, Pinney *et al. *[4] could show an FES rate of only 4%. Analysis of the subgroups showed development of FES manifestations in all patients below the age of 35 as well as in patients in whom treatment had been initiated more than 10 hours after trauma. Our work also reports on a patient under the age of 35, but surgery commenced within six hours of the trauma. The incidence of FES is considerably increased in patients suffering from multiple injuries [2]. In a series of 211 patients suffering from multiple injuries, Riska and Myllynen [5] only found three patients (1.4%) who received surgery; however, one patient died. On the other hand, 84 patients (22%) in the comparison group received conservative treatment. Apart from emerging from fractures [6,7], FES can also be caused iatrogenically by intramedullary nailing of the femur or the tibia. It is assumed that fat particles are introduced into the venous system as a result of increased intramedullary pressure caused by the intramedullary pin, which will almost always result in the formation of droplet-shaped fat agglomerations in the capillary areas of the lungs. This formation will generally lead to pulmonary micro-embolism resulting in increased perfusion pressure, congestion of the lung vessels and secondary overstressing of the right side of the heart, which in turn may result in hypoxemia, probably with acute right-sided heart failure. Furthermore, the bone marrow in the venous vessels causes considerable activation of coagulation with a decrease in thrombocytes and consumptive coagulopathy (disseminated intravascular coagulation). Petechiae (punctuate bleeding) may appear on the trunk of the body as well as sub-conjunctivally as a delayed effect. However, this clinical characteristic was not observed in our patient. The maximum pressure measured during the reaming of the medullary cavity in preparation for a femoral intramedullary pin may reach 400-500 mmHg [8]. These pressure values are primarily achieved during the opening procedure and the first drill sizes. If the medullary cavity is sufficiently widened, the procedure of screwing in the pin will not cause excessive pressures anymore. Screwing the intramedullary pin into an unwidened medullary cavity will lead to pressures of 200-300 mmHg [9]. Here, the screwing process does not cause any increase in pressure; however, screwing in the pin will lead to pressure values as high as those reached during the drilling process. For the prevention of FES, no significant differences were found with regard to the femur, that is whether intramedullary pins were introduced into a widened or an unwidened medullary cavity [10]. Paradoxical FES will occur if the origin is initially located in the venous system, and arterial circulation takes place prior to potential pulmonary manifestation. Potential causes for such manifestations are, for example, latent or patent foramen ovale [11], ventricular septum defects, persistent truncus arteriosus, arteriovenous malformations, or - as in our patient - an atrial septal defect in high position with right-to-left shunting. However, only very few case reports on paradoxical FES are available in the literature. Christie *et al. *[10] reported on four patients with latent foramen ovale, who developed paradoxical FES because of the reaming of the medullary cavity of the femur; two out of these four patients died. The intravasations were documented intra-operatively by means of trans-esophageal echocardiography. Kallina and Probe [12] reported on a 20-year-old female patient with previous mitral valve prolapse, who developed paradoxical FES after fractures of the femur and the tibia. Reaming of the respective medullary cavity was conducted 16 hours after trauma, prior to intramedullary nailing. In contrast to our patient, a decrease of oxygen saturation was noted on the already awake patient at the end of surgery, leading to intubation. Similar to our patient, diagnostic investigation showed cerebral ischemic disorders with white, matt stipples as well as generalized spasticity. In contrast to our patient, this patient was completely oriented again after 55 post-operative days, and speaking did not present a problem to her. Although embolism was not documented intra-operatively by means of echocardiography in our patient, paradoxical cerebral embolism had to be suspected because of the high-positioned atrial septal defect with right-to-left shunting, which had not been diagnosed before. Pulmonary deterioration was not observed at any time, neither diagnostically nor clinically. Finally, the hypothetical question remains whether FES was caused by the femoral fracture itself or by intramedullary nailing. There is evidence indicating that both femur fractures and intramedullary nailing lead to introduction of fat into the circulatory system, not only on their own but also in combination. In our patient, this combination resulted in fulminant paradoxical FES, therefore the authors recommend plating of femoral fractures instead of nailing.

## Conclusion

FES may occur as a fulminant complication of femoral fractures in cases of a concomitant atrial septal defect with a right-to-left shunt. The hypothetical question remains whether FES is caused by the injury itself or by intramedullary nailing. Thus, in patients with cardiac right-to-left shunts, femurs should not be nailed intramedullary, not even in case of an isolated injury.

## Consent

Written informed consent was obtained from the patient for publication of this case report and any accompanying images. A copy of the written consent is available for review by the Editor-in-Chief of this journal.

## Competing interests

The authors declare that they have no competing interests.

## Authors' contributions

MF was a major contributor in writing the manuscript. PC was involved with the acquisition of data. KB and EC were responsible for analyzing the discussion. NM critically revised the manuscript. NC gave final approval of the version to be published. All authors read and approved the final manuscript.
